# Dasatinib inhibits leukaemic cell survival by decreasing PRH/Hhex phosphorylation resulting in increased repression of VEGF signalling genes

**DOI:** 10.1016/j.leukres.2012.07.013

**Published:** 2012-11

**Authors:** Peter Noy, Kevin Gaston, Padma-Sheela Jayaraman

**Affiliations:** a School Immunity and Infection, University of Birmingham, Birmingham, UK; b School of Biochemistry, University of Bristol, Bristol, UK

**Keywords:** Leukaemia, Phosphorylation, BCR-ABL, Transcription

## Abstract

The PRH/Hhex transcription factor represses multiple genes in the VEGF signalling pathway (VSP) to inhibit myeloid cell survival. Protein kinase CK2 phosphorylates PRH and counteracts the inhibitory effect of this protein on cell survival by blocking the repression of VSP genes. Here we show that the BCR-ABL/Src kinase inhibitor dasatinib decreases PRH phosphorylation and increases PRH-dependent repression of Vegf and Vegfr-1. Moreover in the absence of PRH, dasatinib does not inhibit cell survival as effectively as in PRH expressing cells. Thus the re-establishment of gene control by PRH is in part responsible for the therapeutic effects of dasatinib.

## 1 Introduction

Protein kinase CK2 is a constitutive stress-responsive kinase that promotes cell survival and is strongly implicated in tumourigenesis. CK2 activity is elevated in several cancer types including Acute Myeloid Leukaemia (AML) and Chronic Myeloid Leukaemia (CML) [1,2]. CML cells express the oncogenic p210 BCR-ABL fusion protein, a constitutively active tyrosine kinase that stimulates multiple growth promoting signalling pathways. BCR-ABL and Src family kinases interact with each other and both have been shown to increase CK2 activity [3,4]. The CML therapeutic imatinib and its derivative dasatinib inhibit the tyrosine kinase activity of BCR-ABL resulting in leukaemic cell death. Dasatinib is a dual ABL-Src kinase inhibitor that exhibits a more potent but less selective inhibition of BCR-ABL than imatinib and is commonly used in treatment of imatinib resistant CML. Although imatinib and dasatinib are well-characterised treatments for CML the downstream targets for their action are not fully delineated.

The Proline-Rich Homeodomain (PRH/Hhex) protein regulates many processes in embryonic development and in the adult (reviewed [5]). In the haematopoietic system PRH is expressed in myeloid lineages where it functions as a negative regulator of cell growth. Loss of PRH function in myeloid cells contributes to the development of AML subtypes [5]. PRH regulates myeloid survival through the direct transcriptional repression of multiple genes encoding components of the VEGF signalling pathway (VSP) including Vegf, Vegfr-1, Vegfr-2, and neuropillin-1 [6,7]. Phosphorylation of PRH by CK2 inhibits the DNA binding activity of this protein [7] and CK2 antagonises PRH-dependent growth inhibition by altering the stability and activity of PRH (manuscript in revision). Here we show that an important effect of dasatinib is to decrease phosphorylation of PRH resulting in increased PRH repression activity at VSP genes and decreased cell survival.

## 2 Materials and methods

Cell culture, quantitative PCR for Vegf and Vegfr-1 mRNA, PRH knockdown with shRNA PRH or shRNA GFP (control) were performed as described previously [6]. Western blotting was performed with mouse antibodies which preferentially recognise hypophosphorylated PRH and rabbit antibodies that preferentially recognise pPRH [7]. Antibody for phosphoXRCC1 was a kind gift from Dr. Grant Stewart (University of Birmingham). Antibody for pSrc was a kind gift from Dr. Yotis Senis (University of Birmingham). Antibody for pStat5 was obtained from New England Biolabs.

## 3 Results

**Fig. 1:**
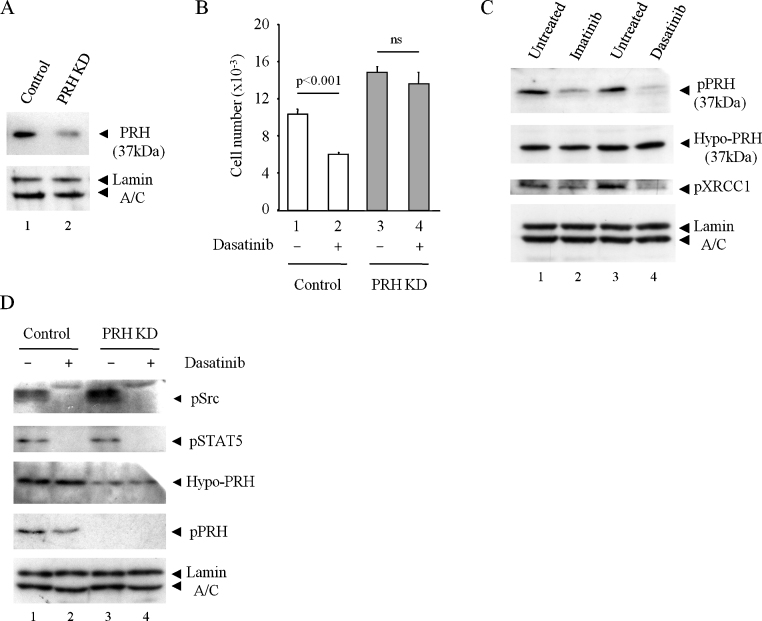
Dasatinib reduces cell survival and decreases PRH phosphorylation. (A) Western blot with mouse polyclonal anti-PRH antibody showing PRH levels in cells selected with puromycin for 10 days following transfection with shRNAGFP (control) or shRNAPRH (PRH KD). Detection of Lamin A/C with laminA/C antibody is used as loading control (B) Control and PRH KD K562 cells were treated with dasatinib (0.5 μM) for 8 h and an MTT assay was then used to determine cell numbers. *M* + SD, *n* = 3. (C) K562 cells were treated with dasatinib (30 μM) or imatinib (100 μM) for 6 h. The extacts were Western blotted for endogenous hypo-PRH or pPRH using mouse polyclonal PRH and rabbit polyclonal phosphoPRH antibodies. The blot was stripped and reprobed for Lamin A/C as a control for protein loading and with a phospho-specific pXRCC1 antibody. (D) Control and PRH KD K562 cells were treated with dasatinib (0.5 μM) for 6 h and then the extracts were probed with antibodies against phosphoSrc (pSrc), phosphoSTAT5 (pSTAT5), endogenous hypo-PRH, phospho-PRH or lamin A/C respectively. Control and knockdown cells treated with dasatinib both show inhibition of BCR-ABL signalling and Src signalling. Only control cells show the reduction in phosphoPRH levels with dasatinib treatment as the levels of pPRH in PRH KD cells is too low to detect.

**Fig. 2:**
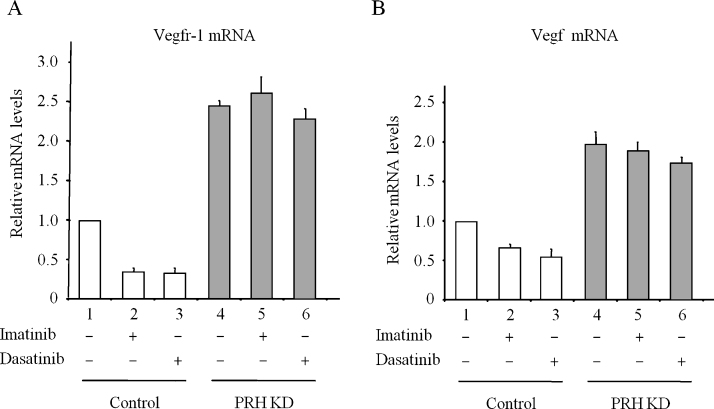
Dasatinib represses VSP gene expression. (A) Control and PRH KD K562 cells were treated with dasatinib as above for 6 h prior to mRNA isolation and qPCR for Vegfr-1 mRNA. *M* + SD, *n* = 3. (B) Vegf mRNA levels in the cells described in (A). *M* + SD, *n* = 3.

**Fig. 3:**
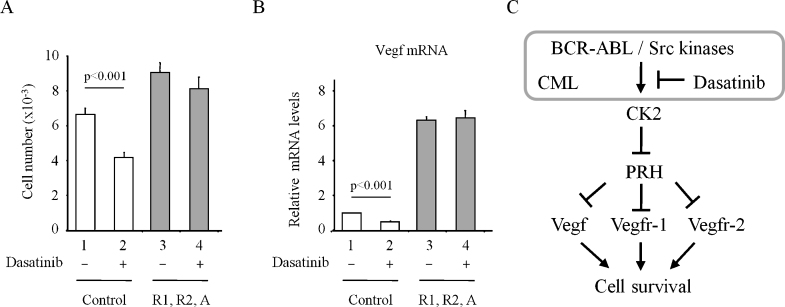
(A) Cells with increased VSP gene expression are resistant to dasatinib treatment. Control and K562 cells over-expressing Vegfr-1, Vegfr-2 and Vegf were treated with dasatinib (0.5 μM) for 8 h and an MTT assay was then used to calculate cell numbers. *M* + SD, *n* = 3. (B) mRNA obtained from cells treated as above was used for quantitative PCR to detect Vegf expression. (C) A model for the misregulation of PRH activity in CML. CK2 inhibits PRH and alleviates the repression of VSP genes resulting in increased cell survival. BCR-ABL increases CK2 activity leading to increased PRH phosphorylation, derepression of VSP genes and increased cell survival. Dasatinib treatment reduces PRH phosphorylation and re-establishes the repression of VSP genes leading to the inhibition of cell survival.

Inhibition of BCR-ABL results in the inhibition of CK2 activity in BCR-ABL-dependent lymphoma cells [4]. K562 cells are a CML cell line that express BCR-ABL and we hypothesised that the inhibition of BCR-ABL in these cells might result in decreased CK2 activity leading to decreased phosphorylation of PRH and consequent reduced cell survival. To test this we generated PRH knockdown cells using shRNA for PRH or shRNA GFP as control (Fig. 1A) as described previously [6]. We treated K562 shRNA control cells and PRH knockdown (KD) cells with the BCR-ABL inhibitor dasatinib. We used a high concentration of dasatinib to ensure that any effect of PRH loss would be relevant to both the on-target effects of this inhibitor (BCR-ABL/Src) and to any off-target effects on other kinases. Importantly CK2 is not a direct target of dasatinib or imatinib. As expected, dasatinib treatment brings about a significant reduction in cell number in control cells (Fig. 1B, 1 and 2) and PRH KD results in increased cell number (Fig. 1B, 1 and 3, respectively). This is because PRH KD is similar to the effect of increased phosphorylation of PRH by CK2. Importantly, treatment of the PRH KD cells with dasatinib has no significant effect on cell number (Fig. 1B, 4). We conclude that PRH plays a significant role in the ability of dasatinib to inhibit K562 cell survival and that both the on-target and any off-target effects of dasatinib treatment are in large part at least dependent on the presence of PRH.

To confirm that dasatinib treatment inhibits K562 cell survival through PRH-mediated repression of VSP genes, we looked at PRH phosphorylation and VSP gene expression. Incubation of K562 cells with high concentrations of dasatinib or imatinib for 6 h results in a decrease in pPRH levels but has no effect on the amount of total PRH (Fig. 1C). In the same experiment phosphorylation of an unrelated CK2 target protein XRCC1 is also reduced confirming that CK2 is inhibited. Similar results were also obtained when cells were treated with 0.5 μM dasatinib for 6 h and extracts were blotted for PRH and pPRH (Fig. 1D). To confirm that this treatment blocks both BCR-ABL activity and Src activity in control and knockdown cells extracts were blotted for pSTAT5 as a down-stream marker of BCR-ABL activity and pSrc. Fig. 1D shows that both BCR-ABL and Src family kinases are strongly inhibited by dasatinib in control cells and in PRH knockdown cells. As expected based on the reduction in pPRH levels (Fig. 1B and 1D), dasatinib and imatinib decrease expression of Vegfr-1 (Fig. 2 A, 2 and 3) and Vegf (Fig. 2B, 2 and 3). Furthermore, when there is little PRH, these drugs have little or no effect on the expression of Vegfr-1 (Fig. 2A, 5 and 6) or Vegf (Fig. 2B, 5 and 6). Finally, we examined whether over-expression of VEGF receptors and VEGF is able to counteract the inhibitory effects of dasatinib on cell survival. The survival of cells over-expressing VEGFR-1, VEGFR-2 and VEGF is not significantly inhibited by dasatinib whereas the survival of control cells is strongly inhibited under the same conditions (Fig. 3 A). Fig. 3B shows that as expected VEGF mRNA levels are indeed elevated in this experiment. This confirms that the effects of dasatinib on these cells are largely mediated by the inhibition of VEGF gene expression.

## 4 Discussion

Blast crisis CML cells have constitutively high BCR-ABL tyrosine kinase activity, elevated VEGF levels that promote angiogenesis and elevated CK2 activity. Imatinib and dasatinib inhibit the tyrosine kinase activity of BCR-ABL and in the case of dasatinib also target other kinases such as the Src family tyrosine kinases, ckit and EphA2, resulting in cell death. Inhibition of these kinases leads to the down-regulation of a variety of signalling pathways including the PI3Kinase pathway and this results in decreased cell survival. We have shown that dasatinb treatment brings about a reduction in PRH phosphorylation. Significantly, the inhibition of K562 cell survival and the decrease in VSP gene expression brought about by dasatinib treatment are largely dependent on the presence of PRH (Fig. 3C). Although dasatinib inhibits multiple kinases, in the absence of PRH even high concentrations of dasatinib have little effect on cell survival. We conclude that the re-establishment of VSP gene regulation by PRH is responsible in large part for the effects of these inhibitors on K562 cells. In agreement with this conclusion de-regulated VSP gene expression is associated with CML [8]. We infer that the elevation of CK2 activity by BCR-ABL signalling [4] very likely results in elevated levels of PRH inactivation in CML. Although CK2 is a pleiotropic kinase CK2 inhibitors are being assessed for the treatment of BCR-ABL transformed ALL and multiple AML where CK2 activity has been found to be elevated [1]. Our results suggest a molecular rationale for the use of CK2 inhibitors in conjunction with BCR-ABL inhibitors in the treatment of primary CML. Experiments in primary cells are required to further investigate the importance of PRH phosphorylation and determine whether the restoration of PRH activity through inhibition of CK2 may be particularly of value in imatinib or dasatinib resistant CML.

## Conflict of interest statement

The authors confirm that there is no conflict of interest.

## References

[1] Kim J.S., Eom J.I., Cheong J.W., Choi A.J., Lee J.K., Yang W.I. (2007). Protein kinase CK2alpha as an unfavorable prognostic marker and novel therapeutic target in acute myeloid leukemia. Clin Cancer Res.

[2] Phan-Dinh-Tuy F., Henry J., Boucheix C., Perrot J.Y., Rosenfeld C., Kahn A. (1985). Protein kinases in human leukemic cells. Am J Hematol.

[3] Donella-Deana A., Cesaro L., Sarno S., Ruzzene M., Brunati A.M., Marin O. (2003). Tyrosine phosphorylation of protein kinase CK2 by Src-related tyrosine kinases correlates with increased catalytic activity. Biochem J.

[4] Mishra S., Reichert A., Cunnick J., Senadheera D., Hemmeryckx B., Heisterkamp N. (2003). Protein kinase CKIIalpha interacts with the Bcr moiety of Bcr/Abl and mediates proliferation of Bcr/Abl-expressing cells. Oncogene.

[5] Soufi A., Jayaraman P.S. (2008). PRH/Hex: an oligomeric transcription factor and multifunctional regulator of cell fate. Biochem J.

[6] Noy P., Williams H., Sawasdichai A., Gaston K., Jayaraman P.S. (2010). PRH/Hhex controls cell survival through coordinate transcriptional regulation of vascular endothelial growth factor signaling. Mol Cell Biol.

[7] Soufi A., Noy P., Buckle M., Sawasdichai A., Gaston K., Jayaraman P.S. (2009). CK2 phosphorylation of the PRH/Hex homeodomain functions as a reversible switch for DNA binding. Nucleic Acids Res.

[8] Janowska-Wieczorek A., Majka M., Marquez-Curtis L., Wertheim J.A., Turner A.R., Ratajczak M.Z. (2002). Bcr-abl-positive cells secrete angiogenic factors including matrix metalloproteinases and stimulate angiogenesis in vivo in Matrigel implants. Leukemia.

